# Andrographolide, a New Hope in the Prevention and Treatment of Metabolic Syndrome

**DOI:** 10.3389/fphar.2017.00571

**Published:** 2017-08-23

**Authors:** Muhammad T. Islam

**Affiliations:** ^1^Department of Pharmacy, Southern University Bangladesh Chittagong, Bangladesh; ^2^Postgraduate Program in Pharmaceutical Sciences, Federal University of Piauí Teresina, Brazil

**Keywords:** cardiovascular diseases, diabetes, dyslipidemia, hypertension, obesity

## Abstract

Recently, the use of plant-derived medicines is increasing interest in the prevention and treatment of a variety of disorders including metabolic syndromes. Metabolic syndrome is one of the major risk factors for cardiovascular diseases (CVDs) and incidence of mortality worldwide. Scientific evidence suggests that *Andrographis paniculata* and its derived components, especially andrographolide (AGL) and its analogs/derivatives have a broad spectrum of biological activities. This review aims to sketch the activity of AGL and its analogs/derivatives against the components of metabolic syndromes such as diabetes, hyperlipidemia, hypertension, and obesity. Additionally, AGL activity against CVDs is also summarized. The finding suggests that AGL and its analogs/derivatives have a potential role in the management of metabolic syndrome; however, more studies should be conducted to evaluate their effectiveness.

## Introduction

According to the National Cholesterol Education Program (NCEP) peoples with at least three out of five have metabolic syndrome (Lakka et al., 2002). The components of metabolic syndrome, such as disturbed glucose and insulin metabolism (high glucose level), overweight and abdominal fat distribution, dyslipidemia, and hypertension are one of the major consequences of CVDs and CVDs-related mortality (Galassi, 2006). To be mentioned that the use of plant-derived compounds has been increased in recent years for the prevention and treatment of various disorders including CVDs (Talha and Priyanka, 2011).

Andrographolide is an extremely bitter C20 labdane diterpenoid, first isolated from *Andrographis paniculata* (Burm. F.) Wall. Ex Nees (Family: Acanthaceae) (Chakravarti and Chakravarti, 1951; Islam, 2016). AGL mainly used for the prevention and treatment of common cold in many countries and known as an anti-inflammatory, antiviral, antithrombotic, hypotensive, and antiatheroscelerotic drug (Amroyan et al., 1999).

The extracts of *A. paniculata* are often used for the treatment of diabetes and other disorders such as inflammatory, cognitive, and psychiatric disorders. Moreover, the AGL has a broad spectrum of therapeutic potential, including metabolic syndrome (Zhou et al., 2013; Thakur et al., 2016). This review depicts the role of AGL in metabolic syndrome mainly the metabolic CVD risk factors including dyslipidemia, high blood glucose, high blood pressure, and obesity.

## Methodology

To gather up-to-date scientific evidence, a search was made in the following databases: *PubMed, Science Direct, Scopus, Web of Science*, and *Google scholar*. No language restriction was imposed. Published evidences (*in vitro, ex vivo*, and *in vivo*) with *A. paniculata* extracts, andrographolide and its analogs and/or derivatives in metabolic syndrome and related diseases have been considered in this study. Reports other than metabolic syndrome, data duplication, and extracts without mentioning andrographolide-enriched are not included in this study.

## Findings

### Effects on High Glucose Level

The chronic metabolic syndrome, diabetes is strictly related to CVDs leading to premature death (Qidwai and Ashfaq, 2014). AGL (orally administered) in streptozotocin (STZ)-induced diabetic rats, decreased the plasma glucose concentrations in a dose-dependent manner, where AGL at 1.5 mg/kg significantly attenuated the increased plasma glucose level in an intravenous glucose challenge test in normal rats. Additionally, in soleus muscle, the messenger-RNA (mRNA) and protein levels of the GLUT4 were found to increase after a repeated intravenous (i.v.) administration (3 days) of AGL in STZ-diabetic rats (Yu et al., 2003). It may be due to an activation of α1-ARs and enhancing the secretion of β-endorphin, thus stimulating the opioid micro-receptors to reduce the hepatic gluconeogenesis and to enhance the glucose uptake in soleus muscle in the animals (Yu et al., 2008).

**FIGURE 1:**
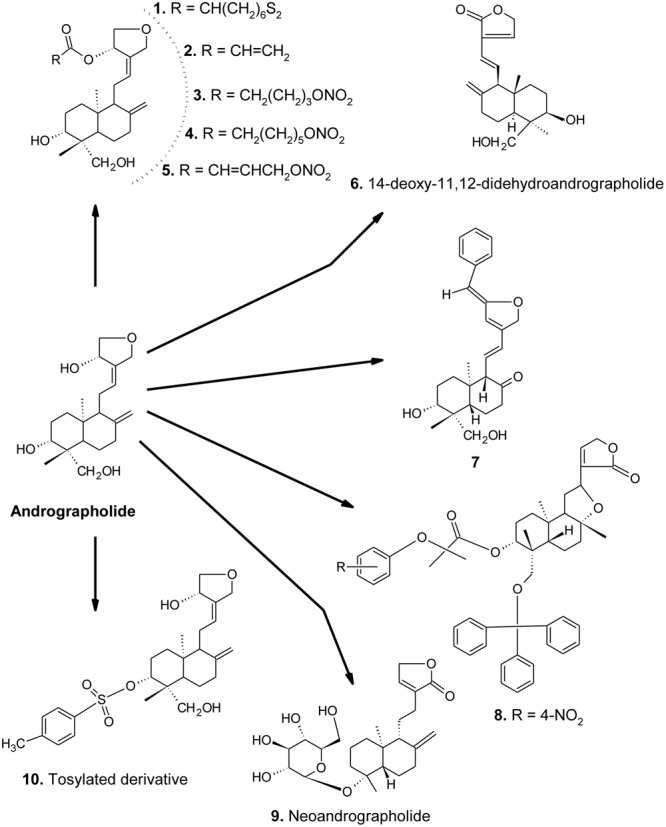
Chemical structures of AGL and its important derivatives acting against metabolic syndrome.

On the other hand, AGL-lipoic acid conjugate [compound **1, Figure 1**, orally (p.o.) once daily for 6 days] in alloxan-treated mice (model: type 1 diabetes) was reported to lower blood glucose, increase in insulin secretion and prevention of loss of β-cells and their dysfunction, stimulating GLUT4 membrane translocation in soleus muscles. Furthermore, pre-treatment of RIN-m cells with compound **1** was found to prevent H_2_O_2_-induced cellular damage, quenching of glucose and glibenclamide-stimulated ROS production, and an inhibition of cytokine-stimulated NF-κB activation (Zhang et al., 2009).

Andrographolide is also evident to increase the glucose uptake in a time- and dose-dependent manner in 3T3-L1 cells. Moreover, AGL suppressed the TNF-α induced activation of NF-κB signaling pathway and its downstream inflammatory factor expression, suggesting a modulatory effect on insulin resistance (Jin et al., 2011). On the other hand, *A. paniculata* extract [2 g/kg/day, intra-gastrically (i.g.)] and AGL (50 mg/kg/day, i.g.) for 5 days in rats were found to increase in protein and mRNA levels and enzyme activities of CYP-2C6/11, -1A1/2, and -3A1/2. Moreover, they also accelerated the tolbutamide metabolic rate, possibly *via* increasing the expression and activity of drug-metabolizing enzymes, without impairing the hypoglycemic effect of tolbutamide (Chen H.W. et al., 2013). AGL or AGL-enriched extract of *A. paniculata* (8 consecutive days) significantly (*p* < 0.05) decreased the levels of blood glucose and improved the neonatal STZ-induced diabetic rats islet’s and beta cells (Nugroho et al., 2014). In the later case, more beneficial effects were observed in AGL-treated group.

### Effects on Diabetes and Diabetic-Related Complications

The ethanolic extract of *A. paniculata* and AGL showed an alpha-glucosidase inhibitory effect in a concentration-dependent manner (IC_50_ = 11.0 to 50.9 mg/mL), suggesting a potential candidate for the management of type 2 diabetes mellitus (Subramanian et al., 2008). Some AGL derivatives (compounds **1–5, Figure 1**) (0.008–5 μM) in *t*-BHP induced RIN-m cell damage were found to inhibit apoptotic cell death (Liang et al., 2014).

Andrographolide and 14-deoxy-11,12-didehydroandro-grapholide (compound **6**) in MES-13, an SV40-transformed murine glomerular mesangial cell line were evident to reduce the phenotypes indicating diabetic nephropathy, where AP2 showed potent activity than AGL in the reduction of apoptosis marker caspase-3, fibrosis marker TGF-β, and PAI*-*1. Moreover, both of them also reduced the intracellular oxidative states in high glucose cultured MES-13 cells (Lee et al., 2010).

In a study, AGL and a water-soluble polysaccharide (galactose: mannose:fucose:arabinose:rhamnose with molar ratios of 15.4:2.5:4.3:1.5:1.6) synergistically decreased the levels of serum creatinine, serum urea nitrogen, urinary albumin excretion, serum urea, and blood glucose in STZ-induced diabetic rats (Xu et al., 2012).

In a study, the methanol extract of *A. paniculata* and AGL were found to decrease galactitol accumulation in rat lens (*in vitro*) and galactosemic rat model (*in vivo*) (Veeresham et al., 2013). Zhou et al. (2013) also introduced two important AGL derivatives (compounds **1** and **7, Figure 1**) having antidiabetic activity.

Andrographolide in STZ-induced DR in C57BL/6 mice was found to ameliorate DR *via* attenuating retinal angiogenesis and inflammation, and VEGF, NF-κB, and Egr1 signaling pathways (Yu et al., 2015).

Moreover, AGL in HepG2 cells probably by binding with adenosine A2α receptor activates Nrf-2 transcription and inhibited its exclusion from the nucleus by inactivating GSK-3β, together resulting in activation of HO-1, suggesting an application in the treatment of diabetes and other related diseases (Mittal et al., 2016). On the other hand, STZ-induced diabetic rats treated with a hydro-methanolic *A. paniculata* leaf extract (50, 100, and 200 mg/kg/day, p.o.), or pure AGL (15, 30, and 60 mg/kg/day, p.o.) for 10 consecutive days, were found to reduce acetylcholinesterase and LPO, while the increase in SOD and CAT activities in the brain tissues in a dose-dependent manner. They also improved diabetic hyperglycemia and insulin deficiency in the experimental animals (Thakur et al., 2016). AGL-induced antidiabetic effect in STZ-induced diabetic rats was also reported by Samala and Veeresham (2016), where AGL altered some pharmacokinetics and pharmacodynamics of parameters of glyburide, such as increased in C_max_, AUC_0_-n, AUC_total_, t_1/2_, and mean residence time while decrease in drug clearance and volume of distribution (Vd) as compared to the control group. There may be an inhibition of CYP-3A4 enzyme.

Diabetic nephropathy is characterized by the proliferation of mesangial cells, mesangial hypertrophy, and extracellular matrix accumulation. AGL (2 mg/kg, i.p., twice a week for 8 weeks) in STZ-induced diabetic C57BL/6 mice decreased in fasting blood glucose, TGs, kidney/body weight ratio, BUN, serum creatinine and 24 h albuminuria. Additionally, AGL also prevented renal hypertrophy and ECM accumulation along with NADPH oxidase 1 (NOX1) expression, production of ROS and pro-inflammatory cytokines (Ji et al., 2016).

### Effects on Blood Pressure

In a study Chen et al. (2014) suggested that, AGL-mediated inhibition of NF-κB activity in TNF-α-stimulated VSMCs may be through JNK-Akt-p65 signaling pathway in an IκBα-independent mechanism. The spray dried AGL formulation for dry powder inhaler in rat model enhanced lung deposition and pulmonary antihypertensive activity (Mali et al., 2016).

On the other hand, water extract of *A. paniculata* (2 g/kg/day, p.o. for a week) in high-fat diet-induced obese mice decreased the myocardial inflammation pathway related proteins that contribute to cardiac hypertrophy and myocardial apoptosis (Hsieh et al., 2016). Moreover, 14-deoxy-11,12-didehydroandrographolide (compound **6**) is known for its potential hypotensive and vasorelaxation effects in rodent models. It has a stronger Ca^2+^ channel blocking capacity than the verapamil (Yoopan et al., 2007).

Moreover, AGL (IC_50_ 5 μM) was found to inhibit platelet-activating factor (PAF)- induced human blood platelet aggregation in a dose dependent manner (Amroyan et al., 1999). In a previous study, Zhang and Tan (1997) suggested that, the crude water extract of *A. paniculata*, its three semi-purified ethyl acetate, *n*-butanol and aqueous fractions, as well as AGL significantly reduced mean arterial blood pressure (MAP) in anesthetized Sprague-Dawley rats between ED_50_ values of 5.0 to 11.4 mg/kg.

### Effects on Lipid Profile

In a study, the synthetic derivatives (30–500 mg/kg, p.o. for 6 days) of AGL were found to reduce the TG, TC and LDL-C, while an increase in HDL-C levels significantly in rodents than the positive control, atromide (Wang et al., 2012). Compound **7** (**Figure 1**) was found more potent than the others. Moreover, AGL was found to inhibit the activation of ERK-1/2, p38, MAPK and NK-κB induced by ox-LDL in macrophage foam cells, suggesting an anti-atherosclerosis activity (Li and Li, 2012).

Andrographolide (10 and 20 mg/kg, p.o.) in hyperlipidemia induced by *Porphyromonas gingivalis* in male Sprague-Dawley rats (*n* = 6) reduced the TC, LDL-C, and TG levels. Additionally, AGL also found to reduce MDA, while an increase in SOD and GPx levels in experimental animals (Al Batran et al., 2013).

In a study, AGL and neoandrographolide (compound **9, Figure 1**) were found to reduce LDL-C, TGs, and TC in high-fat emulsion (75% yolk)-diet mice and rats in a dose-dependent manner. Moreover, the plasma AST and ALT levels were significantly decreased along with the down-regulation of iNOS and up-regulation of eNOS expression (Yang et al., 2013a).

In non-obese diabetic (NOD) mice, AGL (50, 100, and 150 mg/kg, p.o. for 4 weeks) was found to inhibit insulitis, delay the onset, and suppress the development of diabetes in a dose-dependent manner. The protected status was correlated with a substantially decreased production of interferon gamma (IFN-γ) and interleukin (IL)-2 and -17 while increase in IL-10 and TGF-β levels. Furthermore, AGL also increased GATA3 mRNA expression, while a decrease in T-bet and RAR-related orphan receptor gamma t (RORγt) mRNA expressions, suggesting prevention of type 1 diabetes by maintaining Th1/Th2/Th17 homeostasis (Zhang et al., 2013). AGL and its tosylated derivative (compound **10, Figure 1**) (100 mg/kg b.w. in mice) have been also reported for anti-atherosclerosis, anti-dyslipidemic, LDL-oxidation and antioxidant activities (Pandeti et al., 2013).

Andrographolide-loaded solid lipid nanoparticles (average diameter of 286.1 nm) were found to increase in absorption, bioavailability (241%), and anti-hyperlipidemic activity of AGL in Caco-2 cell (Yang et al., 2013b). AGL -enriched extract of *A. paniculata* in high-fat-fructose-fed (36% fructose, 15% lard, and 5% egg yolks in 0.36 g/200 g body weight for 70 days) rat was found to decrease in LDL-C, TC and TGs, while an increase in HDL-C level (Nugroho et al., 2013). Otherwise, in a clinical study, in 60 patients, *A. paniculata* extract, AGL (low dose: 71.64–72.36 mg/day; high dose: 119.64–120.36 mg/day), and gemfibrozil (300 mg/day) were given for 8 weeks, suggesting the high-dose of the extract and AGL significantly reduced the TGs level as compared to the gemfibrozil (Phunikhom et al., 2015).

### Effects on Obesity

In a study, AGL significantly inhibited the adipocyte differentiation as well as adipogenesis-related transcription factor, PPARγ and the expressions of the PPARγ targeted genes, such as cluster of differentiation 36 (CD36), LPL, FAS, and other adipocyte markers in 3T3-L1 preadipocytes (Jin et al., 2012). SREBPs, the major transcription factors that regulate the expression of genes involving biosynthesis of cholesterol, fatty acids, and TGs. AGL in high-fat diet (HFD)-induced obesity C57BL/6 mice found to down-regulating the expressions of SREBPs targeted genes and decreasing the cellular lipid accumulation (*in vitro*). Moreover, AGL at 100 mg/kg/day was also reported to attenuate the HFD-induced body weight gain and fat accumulation in liver and adipose tissues along with an improvement of serum lipid levels and insulin or glucose sensitivity in experimental animals (Ding et al., 2014).

To be mentioned that, oxidative stress plays an important role in lipid storage as well as whole-body energy homeostasis. AGL (10 and 20 μg/mL) in 3T3-L1 pre-adipocytes was found to suppress GPX1 and GSH, suggesting an inhibition of proliferation of these cells (Chen W. et al., 2016). In another study, AGL dose-dependently (0–15 μM) inhibited CCAAT/enhancer-binding protein α (C/EBPα) and C/EBPβ mRNA and protein expression as well as the PPARγ protein level during the adipogenesis of 3T3-L1 cells (Chen C.C. et al., 2016).

### AGL in Cardiovascular Diseases (CVDs)

Aberrant VSMC proliferation and dysfunction in CEC are the two other important causes of CVDs. In a recent study, AGL (20–100 μM) in VSMCs and CECs (isolated from rat artery and mouse brain, respectively) inhibited the cell proliferation, probably *via* reducing the expression of ERK-1/2, and by inhibiting the expression of proliferating cell nuclear antigen (PCNA). AGL also remarkably diminished lipopolysaccharide (LPS)-induced iNOS and cyclooxygenase-2 expression (Chang et al., 2014).

Atherosclerosis is linked with the development of many cardiovascular complications. Abnormal proliferation of VSMCs plays a crucial role in the development of atherosclerosis. The apoptosis of VSMCs, occurring in the progression of vascular proliferation, may provide a beneficial strategy for managing CVDs. In a study, AGL was found to reduce cell viability, possibly *via* inducing apoptosis in VSMCs associated with the ceramide-p47phox-ROS signaling cascade (Chen Y.Y. et al., 2013). Inflammation and endothelial cell dysfunction are thought to be two important initiating events in atherosclerosis. TNF-α, a pro-inflammatory cytokine, induces the expression of cell adhesion molecules and results in monocyte adherence and atheromatous plaque formation. Moreover, AGL in EA.hy926 cells down-regulated TNF-α-induced ICAM-1 expression, possibly *via* attenuating of activation of NF-κB rather than an activation of CREB protein, suggesting a potential cardiovascular-protective capacity (Chao et al., 2011).

The NF-κB transcription factor able to modulate the expression of tissue factor (TF), E-selectin (CD62E) and VCAM-1 is also plugged in the thrombus formation and inflammatory syndrome. AGL in a murine model of arterial restenosis was evident to down-regulate the NF-κB target genes that are critical in thrombosis and inflammation, specific inhibitors of p50, thus, therapeutically valuable for preventing and treating thrombotic arterial and other related diseases (Wang et al., 2007).

Andrographolide (10 and 20 mg/kg, p.o. over 12 weeks) in *P. gingivalis*-induced atherosclerosis male white New Zealand rabbits (*n* = 6) reduced in IL-1β and -6, and CRP. AGL also improved the thickening of atherosclerotic plaques and decreased in alpha-smooth muscle actin (α-SMA) in experimental animals (Al Batran et al., 2014a). In another study, AGL (10 and 20 mg/kg, over 12 weeks) was found to act against liver and renal biochemical changes in atherosclerosis induced by *P. gingivalis* in White New Zealand rabbits. Furthermore, it augmented the SOD, CAT, GPx, GSH, while decreasing in MDA levels in serum. AGL also decreased the level of ICAM-1 and VCAM-1 in experimental animals (Al Batran et al., 2014b).

### Miscellaneous

Abdominal aortic aneurysm, a common aortic disease associated with a high mortality rate is characterized by exuberant inflammation and tissue deterioration. AGL is evident to inhibit the arterial NF-κB activation and reduce the production of pro-inflammatory cytokines [chemokine (C–C motif) ligand (CCL2), C-X-C CXCL10, TNF-α, and IFN-γ] in the rodent model. Moreover, AGL is evident to suppress a α4 integrin expression and attenuated the ability of monocytes/macrophages adhering with the activated endothelial cells (Ren et al., 2016).

Furthermore, AGL (*in vitro*: 25–75 μM cell culture; *in vivo*: 22 and 55 μg/kg in mice) is evident to exert an anti-platelet activity, possibly *via* activation of the eNOS-NO/cGMP pathway, resulting in the inhibition of the PI-3 kinase/Akt-p38 MAPK and PLCγ2-PKC cascades, thereby leading to inhibition of platelet aggregation (Lu et al., 2011).

**FIGURE 2:**
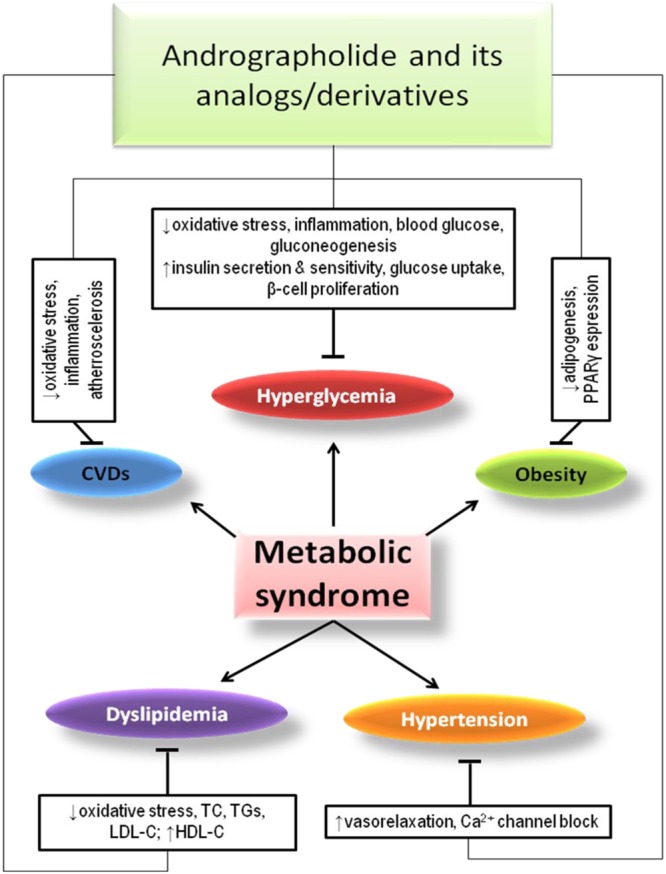
Effects of AGL and its analogs/derivatives on different components of metabolic syndrome.

In a study, *A. paniculata* extract and AGL in high-fructose-fat diet containing 36% fructose, 15% lard, and 5% egg yolks in 0.36 g/200 gb.wt. 55 days induced hyperglycemia in rats, were found to exert hypoglycemic and hypolipidemic effects (Nugroho et al., 2012). In general, the description of protective effects of AGL and its derivatives/analogs against metabolic syndromes has been shown in **Figure 2**.

### AGL-Induced Metabolic Toxicity

In recent years, AGL sodium bisulfite (ASB) has been reported to cause an acute renal failure frequently in clinical practice. Xu et al. (2016) suggest that, ASB at doses 100 or 600 mg/kg/day (for 7 days) increased in protein, occult blood and ketones, where 1,5-anhydroglucitol, D-erythro-sphingosine, and 2-ketoadipate were identified potent influential factors in ASB-induced nephrotoxicity in Sprague-Dawley rats.

## Discussion

Antidiabetic drugs used in the treatment of diabetes mellitus generally act by lowering of blood glucose levels. Lack of insulin is a common consequence of diabetes mellitus type 1. In this case insulin must be must be injected into the patients. On the other hand, insulin resistance of the cells occurs in diabetes mellitus type 2, is the most common type of diabetes. Treatments followed of the diabetes mellitus type 2 include (a) increasing the amount of insulin secretion by the pancreas, (b) increasing the sensitivity of target organs to insulin, and (c) decreasing the rate at which glucose is absorbed from the gastrointestinal tract. AGL is evident to reduce the blood glucose levels by reducing the hepatic gluconeogenesis and enhancing glucose uptake in the experimental animals (Yu et al., 2008; Nugroho et al., 2014).

Andrographolide -lipoic acid conjugate (compound **1**) was found to lower blood glucose, increase in insulin secretion and prevent of loss of β-cells and their dysfunction, stimulate GLUT4 membrane translocation (Zhang et al., 2009), while AGL modulated insulin resistance (Jin et al., 2011), accelerated the metabolic rate, without impairing the hypoglycemic effect of tolbutamide (Chen H.W. et al., 2013), alpha-glucosidase inhibitory effect (Subramanian et al., 2008).

Pancreatic β-cell dysfunction and death is an important feature of diabetes mellitus. Beta-cell protection has demonstrated clinical benefits in the treatment of this disease. AGL and few of its derivatives (Lee et al., 2010; Xu et al., 2012; Zhou et al., 2013; Liang et al., 2014) also demonstrated anti-diabetic effects in some experimental animals.

Aldose reductase (first enzyme in the polyol pathway) catalyzes the reduction of glucose to sorbitol by coupling with the oxidation of NADPH to NADP^+^. An accumulation of sorbitol is evident to various diabetic complications, including neuropathy, nephropathy, cataracts, and retinopathy. Thus, decreasing of galactitol accumulation in rats (Veeresham et al., 2013) and in mice (Yu et al., 2015; Ji et al., 2016) by AGL may be responsible for decreasing of diabetic complications.

Hypertension, the other metabolic risk factor increasing the incidence of a variety of CVDs, such as stroke, coronary artery disease, heart failure, and peripheral vascular disease (Leong et al., 2013). Critical vascular inflammation leads to vascular dysfunction and CVDs, including AAAs, hypertension, and atherosclerosis. AGL-mediated pulmonary antihypertensive activity (Mali et al., 2016), while 14-deoxy-11,12-didehydroandrographolide mediated hypotensive and vasorelaxation effects (Yoopan et al., 2007) in rodent models are also evident nowadays.

Platelet-activating factor is produced in larger quantities by inflammatory cells in response to specific stimuli (Zimmerman et al., 2002). Toxins like fragments of destroying bacteria induce the synthesis of PAF, causing a reduction in blood pressure and reduces volume of blood pumped by the heart, thus leading to shock and even death. AGL inhibition PAF in rats (Zhang and Tan, 1997) and in human (Amroyan et al., 1999) is a good indication for its anti-hypotentive and related other events.

High triglycerides (TGs) and low HDL-C levels are two common consequences risk factors in CVDs (Ahmad and Beg, 2013). On the other hand, obesity, especially excess abdominal fat is a great risk factor for heart diseases (Datau et al., 2010). Thus, the overall findings on AGL, *A. paniculata* extracts as well as AGL derivatives-mediated hypolipidemic effect may be helpful to manage hyperlipidemia and its related events.

Obesity, a medical condition which is characterized by an excess body fat and increased the risk of various diseases and conditions such as CVDs, type 2 diabetes, obstructive sleep apnea, certain types of cancer, osteoarthritis, depression, and so on (Luppino et al., 2010). It is evident that AGL has anti-obesity effects in a number of experimental animals (Jin et al., 2012; Ding et al., 2014; Chen C.C. et al., 2016; Chen W. et al., 2016).

Cardiovascular disease is a class of diseases involving the heart or blood vessels. More common CVDs include coronary artery diseases such as angina and myocardial infarction (also known as a heart attack), stroke, heart failure, hypertensive heart disease, rheumatic heart disease, cardiomyopathy, heart arrhythmia, congenital heart disease, valvular heart disease, carditis, aortic aneurysms, peripheral artery disease, thromboembolic disease, and venous thrombosis (Mendis et al., 2011; GBD 2013 Mortality and Causes of Death Collaborators, 2015). Findings from the literature survey suggest that AGL and its few derivatives were found to protect the cardiac system in a number of experimental animals through a number of molecular mechanisms. Moreover, AGL and its derivatives were evident to reduce oxidative stress and inflammatory reactions in rodents and other test systems. Except ASB there is a lack of toxicity evidence on AGL and its other derivatives. Therefore, more researches are necessary on AGL and its derivatives.

## Conclusion

This review summarized a variety of studies conducted on AGL, AGL-enriched *A. paniculata* extracts and their derived components to find out their roles in metabolic syndromes. Findings suggest that, AGL and its derivatives/analogs have beneficial effects in different components of metabolic syndrome, including diabetes, dyslipidemia, hypertension, and obesity. Thus, AGL may be considered as one of the potential therapeutic tools in the prevention and treatment of metabolic syndromes.

## Author Contributions

The author confirms being the sole contributor of this work and approved it for publication.

## Conflict of Interest Statement

The author declares that the research was conducted in the absence of any commercial or financial relationships that could be construed as a potential conflict of interest.

## Abbreviations

AAA: abdominal aortic aneurysm
AGL: andrographolide
ALT: alanine transaminase
AST: aspartate transaminase
BUN: blood urea nitrogen
C/EBPα: CCAAT/enhancer-binding protein α
CAT: catalase
CCL2: chemokine (C–C motif) ligand
CD36: cluster of differentiation 36
CEC: cerebral endothelial cell
cGMP: cyclic guanosine monophosphate
COX: cyclooxygenase
CREB: cAMP response element binding protein
CRP: C-reactive protein
CVDs: cardiovascular diseases
CXCL10: C-X-C motif chemokine 10
DR: diabetic retinopathy
eNOS: endothelial NOS
ERK: extra-cellular receptor kinase
GATA3: GATA binding protein 3
GLUT4: glucose transporter subtype 4
GPx: glutathione peroxidase
GSH: reduced/total glutathione
HDL-C: high-density cholesterol
HO-1: heme oxygenase 1
ICAM-1: intercellular adhesion molecule 1
IFN-γ: interferon gamma
IL: interleukin
iNOS: inducible nitric oxide synthase
LDL-C: low-density cholesterol
LPL: lipoprotein lipase
LPO: lipid peroxidation
MAPK: mitogen-activated protein kinase
MDA: malondialdehyde
NF-κB: nuclear transforming factor kappa B
NOX1: NADPH oxidase 1
PAI*-*1: plasminogen activator inhibitor type 1
PI3K: phosphotidylinositol 3 kinase
PPARγ: peroxisome proliferator-activated receptor γ
ROS: reactive oxygen species
SOD: superoxide dismutase
SREBPs: sterol regulatory element-binding proteins
STZ: streptozotocin
*t*-BHP: *tert*-butyl hydroperoxide
TC: total cholesterol
TGF-β: transforming growth factor beta
TGs: triglycerides
TNF-α: tumor necrosis factor alpha
VCAM-1: vascular cell adhesion molecule-1
VEGF: vascular endothelial growth factor
VSMCs: vascular smooth muscle cells
